# Space–Time Relationship between Short-Term Exposure to Fine and Coarse Particles and Mortality in a Nationwide Analysis of Korea: A Bayesian Hierarchical Spatio-Temporal Model

**DOI:** 10.3390/ijerph16122111

**Published:** 2019-06-14

**Authors:** Dayun Kang, Yujin Jang, Hyunho Choi, Seung-sik Hwang, Younseo Koo, Jungsoon Choi

**Affiliations:** 1 Department of Applied Statistics, Hanyang University, Seoul 04763, Korea; dayun4927@hanyang.ac.kr (D.K.); jennyujin@hanyang.ac.kr (Y.J.); gusgh4950@hanyang.ac.kr (H.C.); 2 Department of Public Health Sciences, Graduate School of Public Health, Seoul National University, Seoul 08826, Korea; cyberdoc@snu.ac.kr; 3 Department of Environmental Engineering, Anyang University, Anyang 14028, Korea; koo@anyang.ac.kr; 4 Department of Mathematics, Hanyang University, Seoul 04763, Korea; 5 Research Institute for Natural Sciences, Hanyang University, Seoul 04763, Korea

**Keywords:** air pollution, mortality, cardiovascular disease, respiratory disease, spatio-temporal model, Poisson model, Bayesian approach

## Abstract

Previous studies have shown an association between mortality and ambient air pollution in South Korea. However, these studies may have been subject to bias, as they lacked adjustment for spatio-temporal structures. This paper addresses this research gap by examining the association between air pollution and cause-specific mortality in South Korea between 2012 and 2015 using a two-stage Bayesian spatio-temporal model. We used 2012–2014 mortality and air pollution data for parameter estimation (i.e., model fitting) and 2015 data for model validation. Our results suggest that the relative risks of total, cardiovascular, and respiratory mortality were 1.028, 1.047, and 1.045, respectively, with every 10-µg/m^3^ increase in monthly PM_2.5_ (fine particulate matter) exposure. These findings warrant protection of populations who experience elevated ambient air pollution exposure to mitigate mortality burden in South Korea.

## 1. Introduction

Air pollution has been viewed as a threat to human health since the onset of rapid industrialization. It is widely known that air pollutants, including particulate matter with an aerodynamic diameter less than 10 µm (PM_10_) or 2.5 µm (PM_2.5_; fine particulate matter), is linked to total, cardiovascular, and respiratory mortality [1]. Since air pollution is particularly detrimental to vulnerable people, such as senior citizens and infants, understanding how ambient air pollution affects health is very important for making relevant policy [2,3]. Several studies have been conducted regarding the association of PM_10_ and health in South Korea. One study [3] showed that post-neonatal infants are most susceptible to PM_10_ in terms of mortality, especially respiratory mortality. Post-neonatal mortality increased by 14.2% for each 42.9-µg/m^3^ rise in PM_10_. Another study [4] showed that with a 43.12-µg/m^3^ increase in PM_10_ concentration, daily non-accidental, respiratory, cardiovascular, and cerebrovascular mortality increased by 3.7%, 13.9%, 4.4%, and 6.3%, respectively. 

Numerous epidemiological studies have investigated the associations of PM_10_ and PM_2.5_ with mortality worldwide [5,6,7,8,9]. One study [5] concluded that PM_10_ concentration is associated with total, cardiovascular, and respiratory daily mortality in 16 cities in China using a Poisson regression model. Another study [6] showed how air pollutants affect human health in 25 European cities across 12 countries. They determined that a decrease in PM_2.5_ level could have led to a gain in life expectancy, postponing many deaths in the city. A European study [7] determined that the daily total mortality rose as PM_10_ and black smoke concentrations increased with a Poisson regression model. The study [8] investigated the short-term positive effects of PM_10_ and PM_2.5_ on all-cause, cardiorespiratory, and other-cause mortality in the United States using a Bayesian hierarchical model. Another study in the United States [9] showed that increases in PM_10_ and PM_2.5_ concentrations led to increased numbers of post-neonatal infant deaths in 96 cities with populations greater than 250,000. Most of the domestic and foreign studies mentioned above focused on the effects of air pollutants on health outcomes in specific locations. However, to establish national-level air pollution control policy, a study should consider the entire spatial domain of a country. 

Spatio-temporal modeling of the association between air pollution and health has recently received attention in environmental epidemiological studies [10,11]; however, there are some challenges in this domain. First, there is frequently a misalignment problem, due to the different dataset sources. This is because air pollution monitoring data are collected at monitoring stations, while health data are collected at an aggregated areal level. There are several approaches to overcome this problem. For example, after spatial modeling of air pollution data, spatial kriging can be conducted, and areal-level air pollution estimates can be produced [12,13,14,15]. Alternatively, one study [16] proposed a Markov Cube Kriging method to improve computational efficiency. In this paper, we applied a commonly used numerical interpolation method to obtain areal-level air pollution data. Second, there is a spatial confounding bias because space-time varying air pollution covariates and space-time random effects are simultaneously included in space-time health modeling [17]. For example, air pollution may be related to space-time random effects, which would bias the estimate on the effects of air pollution. In this paper, to better estimate air pollution effects without the influence of spatial confounding bias, we adopted the two-stage model proposed in [15].

Until now, most of the epidemiologic studies of air pollution conducted in South Korea have focused on how temporally varying air pollutant concentrations affected mortality in individual cities [18,19], but such studies should be conducted over entire areas of Korea to establish national-level air pollution control policy for health improvement. No studies have examined the relationship between temporally varying air pollutants and mortality throughout South Korea. Additionally, there have been almost no studies on the link between mortality and PM_2.5_ in South Korea [18]. Herein, we investigated the associations of PM_10_ and PM_2.5_ with mortality throughout South Korea from 2012 to 2015. A two-stage Bayesian spatio-temporal hierarchical model was employed to better estimate the effects of air pollution on mortality outcomes, as well as to better predict the mortality associated with ambient air pollution. 

## 2. Data and Methods

### 2.1. Study Domain

**Figure 1:**
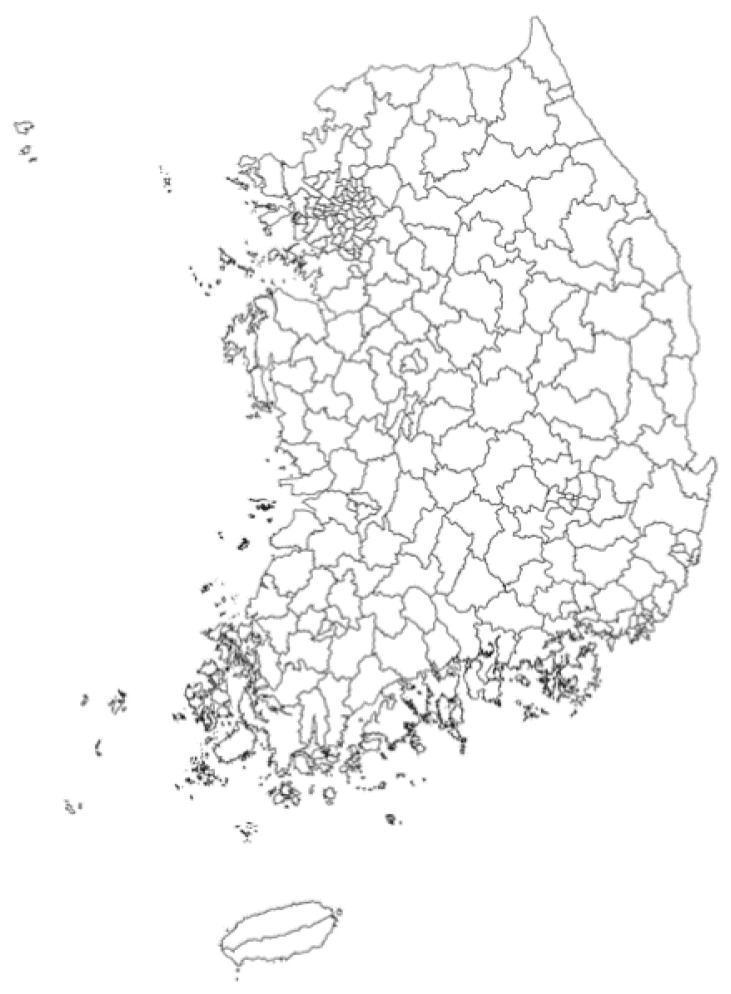
Map of administrative districts in South Korea as of 2012.

This study was conducted over the entire area of South Korea and accounted for the period from 2012 to 2015. There are 250 administrative districts in South Korea (Figure 1) and, because some administrative districts changed during the study period, we conducted analyses based on the administrative areas as of 2012. We used the data from 2012–2014 for model fitting, and data from 2015 for forecasting and evaluation.

### 2.2. Data Description 

We obtained monthly mortality data for 250 administrative districts using the Micro Data Integrated Service (MDIS) from Statistics Korea [20]. We used three types of mortality data: total mortality (all deaths except ICD codes V01–Y98), cardiovascular mortality (I00–I99), and respiratory mortality (J00–J99).

We acquired monthly average concentrations of PM_10_ and PM_2.5_ and meteorological data (temperature, humidity, and wind speed) for 250 districts for the years 2012–2015. The district-level air pollution data were produced based on Pun’s interpolation method [21], a data-assimilation method that combines data from monitoring stations and numerical model outputs.

The regional deprivation index (RDI) was also added to the covariates to control for socio-economic status effects. Because there was little temporal variation in the regional deprivation index, we only used data from 2010. Higher index values indicated that the area was more economically deprived. Because deaths occur more often in areas with relatively large elderly populations, we used the number of people over the age of 65 as an offset. Table 1 shows the summary statistics for the 36 months of data (2012–2014) and 250 administrative districts.

### 2.3. Statistical Analysis

We proposed a two-stage Bayesian hierarchical spatio-temporal model (Model 3) to capture the spatial and temporal dynamics. Two competing models (Model 1, Model 2) were used to compare performances of the proposed model, Model 3. Mathematical expressions for Model 1 and Model 2 are as follows:

**Model 1:**$\log\left(\theta_{it}\right)=\beta_{0}+\beta_{1}X_{it}+S_{1}\left(Z_{it}\right)+S_{2}\left(W_{it}\right)+S_{3}\left(Q_{it}\right)+\delta D_{i}$,
**Model 2**: $\log\left(\theta_{it}\right)=\beta_{0}+\beta_{1}X_{it}+S_{1}\left(Z_{it}\right)+S_{2}\left(W_{it}\right)+S_{3}\left(Q_{it}\right)+\delta D_{i}+u_{i}+v_{i}+k_{t}+l_{t}+\phi_{it}$.

The observed mortality for area i and month t, $y_{it}$, followed a Poisson distribution with mean $\theta_{it}N_{it}$, where $\theta_{it}$ is the relative risk, and $N_{it}$ is the elderly population. The constant term $\beta_{0}$ indicates the intercept of the log of the relative risk that was common to all areas and months. Covariates $X_{it},\;Z_{it},\;W_{it}$, and $Q_{it}$ are the monthly average air pollutant concentrations of ${\mathrm{PM}}_{10}$ or ${\mathrm{PM}}_{2.5}$, temperature, humidity, and wind speed, respectively. The socio-economic covariate $D_{i}$ indicates the deprivation index. The smoothing function $S_{i}\left(\cdot \right),\;i=1,2,3$ denotes a natural cubic spline function to explain the nonlinear effects of meteorological variables on mortality. The degrees of freedom of the natural cubic spline were 3, 2, and 3 for temperature, humidity, and wind speed, respectively. The parameters $\beta_{1}\;\mathrm{and}\;\delta$ denote the regression coefficients $X_{it}$ and $D_{i}$, respectively. The random effects $u_{i}$ and $v_{i}$ are spatially uncorrelated and correlated terms, respectively, and $k_{t}$ and $l_{t}$ are temporally uncorrelated and correlated terms. Lastly, the random component $\phi_{it}$ is the space-time interaction term. 

To deal with the spatial confounding bias problem [17] in Model 2, a two-stage model [22] was considered. In the first stage, the Poisson regression model with only covariates (Model 1) was used. Using this model, we acquired the estimated relative risk $\hat{\theta_{it}}$, and the continuous-type residuals $\hat{r_{it}}$ were obtained from

(1) $$\hat{r_{it}}=\log\left(\frac{y_{it}}{N_{it}}\right)-\log(\hat{\theta_{it}})$$

To capture the extra spatial and temporal variations in the residuals, we considered the following model:

(2) $$\hat{r_{it}}|S_{it}\sim N\left(S_{it},\sigma_{r}^{2}\right)$$

(3) $$S_{it}=u_{i}+v_{i}+k_{t}+l_{t}\;+\phi_{it}$$

In the second stage, our model was expressed as follows, using $\hat{S_{it}},$ the estimated $S_{it}$:

**Model 3:** $\log\left(\theta_{it}\right)=\beta_{0}+\beta_{1}X_{it}+S_{1}\left(Z_{it}\right)+S_{2}\left(W_{it}\right)+S_{3}\left(Q_{it}\right)+\delta D_{i}+\hat{S_{it}}.$

Regression coefficient estimation was performed at this stage.

In the Bayesian framework, we used non-informative prior distributions for the parameters. For the intercept $\beta_{0}$ and air pollutant coefficient $\beta_{1}$, we assumed normal distribution with zero 0 and variance 1,000,000, which is a fairly flat prior. For random effects, spatially and temporally uncorrelated terms had independent and identical normal distributions with zero mean hyperparameters $\sigma_{u}$ and $\sigma_{k}$. The spatially correlated random term $v_{i}$ had a conditional autoregressive (CAR) [23] prior, and the temporally correlated random term $l_{t}$ had an autoregressive (AR)(1) prior. For the interaction term $\phi_{it}$, we considered four of the types proposed in [24]. The interaction term with different spatial trends for each time unit showed the best performance. Uniform distributions with lower bound 0 and upper bound 10 were specified for all hyperparameters.

The three models described above were fitted for the 2012–2014 data. Based on the results from the fitted models, we forecasted death counts for 2015 for all administrative regions in South Korea. We followed the forecasting scheme used in [25].

Bayesian analyses were carried out using the WinBUGS statistical package [26] Two parallel Monte Carlo Markov Chains (MCMC) were used with different initial values. To assess sample convergence, we utilized trace plots, auto-correlation plots, and the Gelman–Rubin statistic [27]. After burn-in, we generated 2500 samples for each chain with thin 50, resulting in a total of 5000 posterior samples. Including the burn-in period, it took around 30 hours to obtain 5000 posterior samples with a CPU Intel Xeon gold 5118 2.3 GHz and RAM 32 GB computer. The open source software R [28] was used to produce the figures in this paper.

We compared the performance of Models 1–3 to identify the model with the best performance, which is shown in Table 2. For comparison, the deviance information criterion (DIC) and mean squared prediction error (MSPE) were used to evaluate model fitness in the Bayesian framework and prediction performance, respectively. The mathematical expression for MSPE is as follows:

(4) $$MSPE=\frac{1}{N}\sum_{i=1}^{I}\sum_{t=1}^{T}{\left(y_{it}-\hat{y_{it}}\right)}^{2},$$

where $y_{it}$ is the observed value, and $\hat{y_{it}}$ is the predicted value. DIC is defined as follows:

(5) $$DIC=pD+\bar{D\left(\alpha \right)},$$

where $\bar{D\left(\alpha \right)}$ is the posterior mean of deviance D(α)=−2logf(y|α), and pD is $\bar{D\left(\alpha \right)}-D\left(\hat{\alpha }\right)$, where $\hat{\alpha }$ is the posterior mean of the parameter α.

To investigate the impact of the degrees of freedom in the spline functions of meteorological variables, we performed a sensitivity analysis by changing degrees of freedom from 2 to 12. The models differed very little in terms of regression coefficient estimates of air pollution and model performance. 

## 3. Results

**Figure 2:**
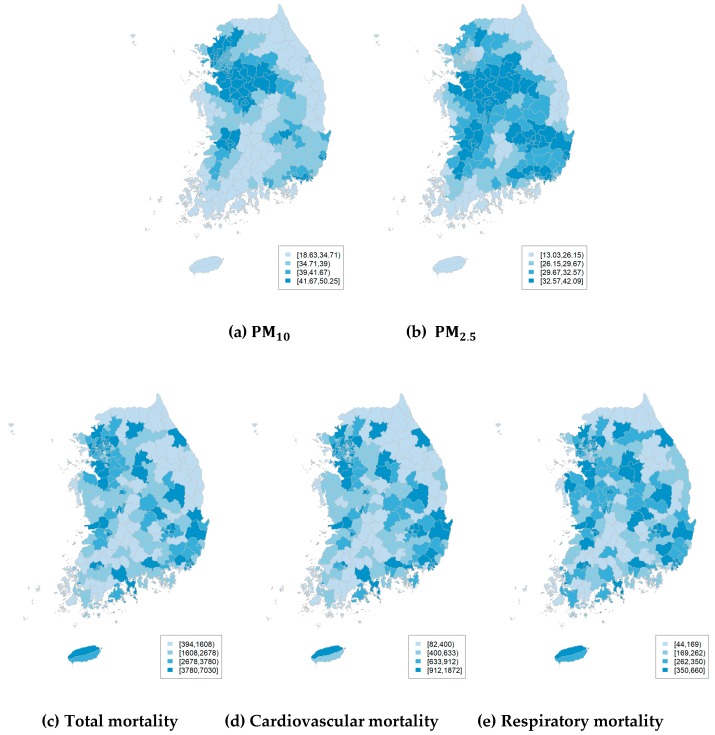
Spatial distributions of air pollutants and mortality. (**a**) and (**b**) are monthly averages of air pollutants from 2012 to 2014; (**c**), (**d**), and (**e**) are monthly sums of deaths from 2012 to 2014.

To show the spatial distributions of air pollutants and mortality in South Korea over the period of 2012–2014, we used the average values of air pollutant concentrations for 36 months and the total death counts for each region over the 36-month period, which are presented in Figure 2. Each map is partitioned into four colors based on quantile. As shown in Figure 2, the total number of deaths from 2012 to 2014 was highest in Nowon-gu, in Seoul, at 7030, and lowest in Gyeryong-si, in Chungcheongnam-do, at 394. Moran’s I statistic was applied to measure the spatial dependencies of mortality and air pollution data at the administrative region level. The null hypothesis of Moran’s I statistic is that there is no spatial correlation among the data. As the adjacency matrix from Moran’s I statistic, various neighborhood structures including binary, row-standardized, and globally standardized techniques were considered. Binary neighborhood structure used a matrix with a value of 1 if it was adjacent, or 0 otherwise. Row-standardized neighborhood structure was used to divide the binary matrix by the sum of the rows. Globally standardized neighborhood structure was used to divide the binary matrix by the mean of the sum of the rows. Moran’s I statistics using row-standardized neighborhood structure of the total, cardiovascular, and respiratory mortality data were 0.27, 0.25, and 0.14, respectively. The results of Moran’s I test for all neighborhood structures showed that all mortality data had statistically significant spatial dependency, with a p-value less than 0.05. Similarly, Moran’s I test indicated spatial variations in ${\mathrm{PM}}_{10}$ and ${\mathrm{PM}}_{2.5}$ with p-values less than 0.05.

Model performance for total mortality and ${\mathrm{PM}}_{10}$ is shown in Table 2. Smaller MSPE and DIC values indicate a better model. While the difference in MSPE between Model 2 and Model 3 was not large, Model 3 yielded a smaller DIC than the other models. Therefore, Model 3 was chosen as the best model. Based on these results, we fitted only Model 3 for cardiovascular and respiratory deaths.

**Figure 3:**
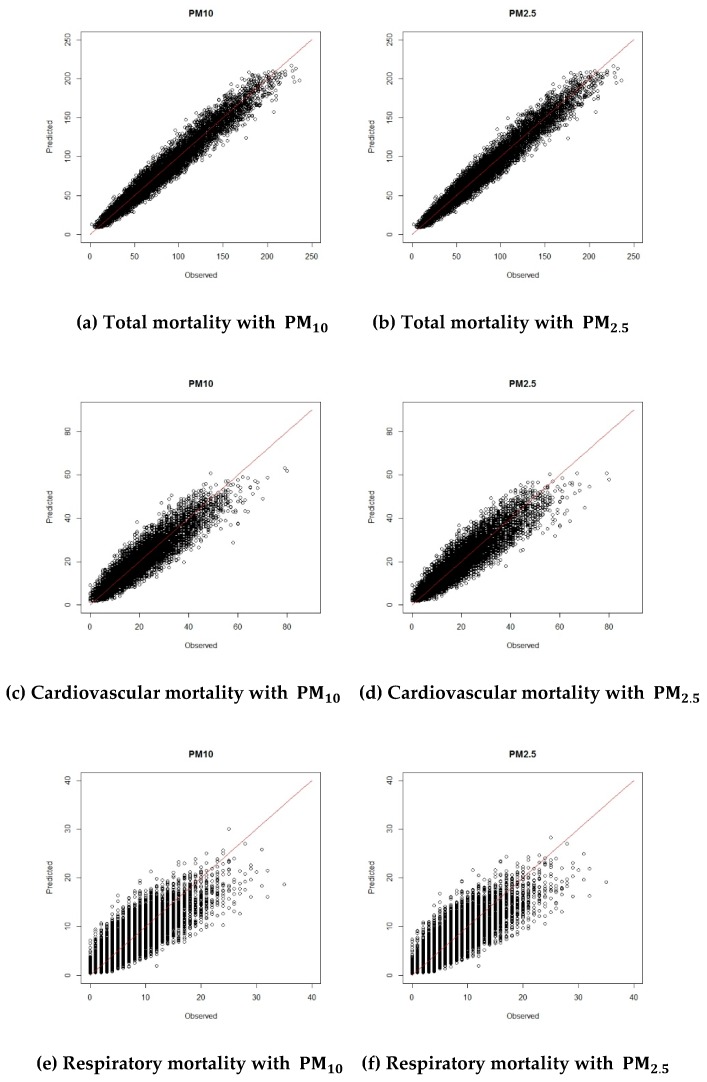
Calibration plots for the observed and estimated values of 2012–2014 using Model 3.

**Figure 4:**
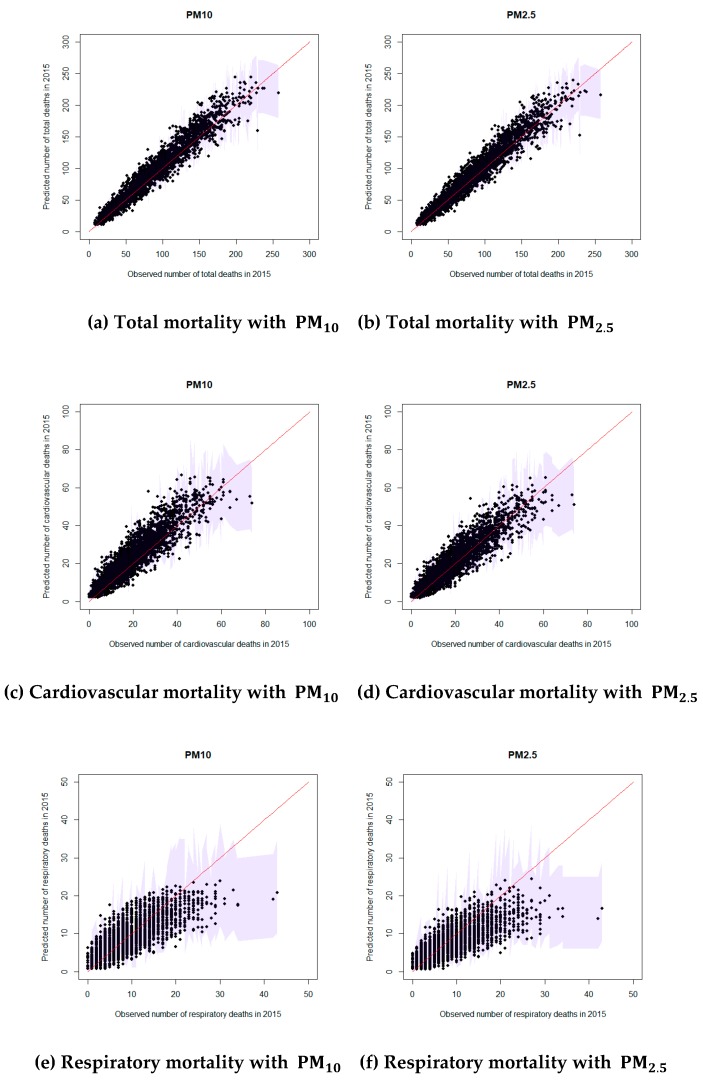
Calibration plots for the observed and forecasted values of 2015 using Model 3.

Table 3 gives the posterior summaries of the estimated coefficients of the explanatory variables. The relative risks of air pollutants are based on a 10-μg/m^3^ increase. The estimated relative risks (RR) of ${\mathrm{PM}}_{10}$ and deprivation index for total mortality were 1.011 and 1.001, respectively. Since 95% credible intervals of all variables did not include 1, all estimated coefficients except ${\mathrm{PM}}_{10}$ for respiratory mortality were statistically significant. When the relative risk was larger than 1, we inferred that the number of deaths increases as the corresponding variable increases. Figure 3 shows the calibration plots of the observed and estimated numbers of deaths for 2012–2014 for the best model, Model 3. The points are close to the line y=x (red line in Figure 3), indicating that the estimation matched the observations well. Figure 4 shows the calibration plots for the observed and forecasted death counts in 2015 using Model 3 with the 2012–2014 data. It shows that the forecasted numbers of deaths were very similar to the observed numbers of deaths. To see the forecasting performance in detail, we calculated the Pearson residuals of the observed and forecasted values, which are shown in Figure S1 in the supplementary material.

## 4. Discussion 

This study focuses on verifying the association between air pollution and human health over the entire area of South Korea for the years 2012–2015. To adjust for other effects on mortality, we also used meteorological factors and regional deprivation index as covariates. 

After controlling for the effects of weather variables, the deprivation index was positively associated with different types of mortality. A previous study [19] found that socioeconomic status, as represented by marital status, education level, and occupation, was linked to different causes of death. Because the deprivation index is a comprehensive index encompassing all types of social or economic statuses for small geographic areas, areas with higher values for this index represent areas with greater deprivation. Deprived areas generally have higher death rates; therefore, our findings are consistent with those of [19].

After controlling for other factors, increases in the concentrations of ${\mathrm{PM}}_{10}$ and ${\mathrm{PM}}_{2.5}$ were associated with increases in various types of mortality. Other than the non-significant association of ${\mathrm{PM}}_{10}$ with respiratory death, these findings are consistent with those from several previous studies [29,30,31]. One previous study [29] determined that the daily ${\mathrm{PM}}_{10}$ concentration was positively associated with daily mortality. One of their findings was that ${\mathrm{PM}}_{10}$ was positively associated with all-cause, cardiovascular, and respiratory mortalities after adjusting for weather variables [29]. Although there are very few existing studies addressing the relationship between ${\mathrm{PM}}_{2.5}$ and death in South Korea, the effects of ${\mathrm{PM}}_{2.5}$ on mortality can be inferred from previous studies on ${\mathrm{PM}}_{10}$ and human health.

One of our study’s strengths is that it covered the entire area of South Korea from 2012 to 2015. Although some studies have considered different geographic areas in South Korea, most did not evaluate spatial and temporal dynamics. For instance, [18] focused on the seven metropolitan cities in South Korea, but analyzed each geographic area independently. In contrast, our study area encompassed all 250 South Korean administrative areas, and we also considered spatial and temporal variations. We utilized spatial and temporal random effects to capture the spatial and temporal dependency structures not captured by the covariates. As far as we are concerned, this study is the first attempt to illustrate the link between air pollutants and human health using a Bayesian two-stage spatio-temporal model.

Despite the many strengths of this study, there are several limitations. First, the regression coefficient of particulate matter is the same for all administrative areas in our proposed model. However, since the effect of air pollution on death might vary across regions, region-specific regression coefficients may be more suitable. This will be one of the future directions of our research into air pollution and human health. Second, stratification of age and sex might lead to a more detailed understanding of air pollution and health. Several previous studies have conducted age- and sex-specific analyses and obtained meaningful findings [5]. Lastly, the results of this paper may be biased due to the possibility of exposure misclassification resulting from the aggregated nature of the data. For example, we assigned air pollution averages to all cases in each district for a given month, which may not truly capture the monthly average exposure for the 30 days prior to the day of mortality. Ideally, individual level data with precise location and date of death are needed to assess daily time-lagged effects of air pollution exposure [32,33,34]. Future research should be geared to address the above limitations.

## 5. Conclusions

In conclusion, this study contributes to an understanding of the relationship between air pollution and human health using a Bayesian two-stage spatio-temporal model. Our findings show that air pollution positively affects total, cardiovascular, and respiratory mortalities. Additionally, this study indicates the need for considering spatio-temporal dynamics in epidemiological studies.

## Figures and Tables

**Table 1:** Summary of variables (2012–2014).

Type	Variable	Unit	Mean	SD	Med	Q1	Q3	Min.	Max
Mortality	Total	person	78.8	43.2	73	44	106	2	236
	Cardiovascular	person	19.3	11.4	17	11	26	0	80
	Respiratory	person	7.7	4.8	7	4	10	0	35
Air pollutant	PM_10_	µg/m^3^	37.7	12.1	36.8	28.8	46	3.7	83.2
	PM_2.5_	µg/m^3^	29.4	10.5	28.3	21.5	36.7	1.6	69.6
Meteorological data	Temperature	°C	13.2	9.6	14.1	5.2	21.7	−7.9	29.7
	Humidity	%	71	9	71.1	64.6	78.1	44.2	92.6
	Wind speed	m/s	2.9	0.9	2.7	2.3	3.3	1.3	8.2
Extra data	RDI		−0.1	8.4	−1.3	−6.8	6.6	−22.5	16.5
	Population	person	202,196	154,313	170,220	60,151	308,111	18,036	669,068

RDI: Regional deprivation index, Med: median, Q1: first quantile, Q3: third quantile.

**Table 2:** Model performance.

Model	MSPE	Deviance	pD	DIC
Model 1	184.22	72,690	11	72,701
Model 2	77.09	63,597	548	64,145
Model 3	79.25	63,098	10	63,108

MSPE: Mean squared prediction error, pD: the effective number of parameters, DIC: Deviance information criterion.

**Table 3:** Relative risks with 95% credible intervals for mortality in Model 3.

Air pollutant	Explanatory Variables	Total	Cardiovascular	Respiratory
		Mean (95% CI)	Mean (95% CI)	Mean (95% CI)
PM_10_	Air pollutant	1.011 (1.008, 1.013)	1.014 (1.009, 1.019)	0.998 (0.991, 1.006)
	Deprivation index	1.001 (1.001, 1.002)	1.003 (1.002, 1.004)	1.016 (1.015, 1.017)
PM_2.5_	Air pollutant	1.028 (1.026, 1.031)	1.047 (1.042, 1.053)	1.045 (1.036, 1.053)
	Deprivation index	1.001 (1.001, 1.001)	1.003 (1.002, 1.004)	1.016 (1.015, 1.017)

## Supplementary Materials

The following are available online at https://www.mdpi.com/1660-4601/16/12/2111/s1, Figure S1: Plots of Pearson residuals versus the forecasted values for different types of mortality and air pollutants.

## References

[1] Samet J.M., Dominici F., Curriero F.C., Coursac I., Zeger S.L. (2002). Fine particulate air pollution and mortality in 20 US cities, 1987–1994. N. Engl. J. Med..

[2] Kwon H., Cho S., Chun Y., Lagarde F., Pershagen G. (2002). Effects of the Asian dust events on daily mortality in Seoul, Korea. Environ. Res..

[3] Ha E., Lee J., Kim H., Hong Y., Lee B., Park H., Christiani D. (2003). Infant susceptibility of mortality to air pollution in Seoul, South Korea. Pediatrics.

[4] Kim H., Kim Y., Hong Y. (2003). The lag-effect pattern in the relationship of particulate air pollution to daily mortality in Seoul, Korea. Int. J. Biometeorol..

[5] Chen R., Kan H., Chen B., Huang W., Bai Z., Song G., Pan G. (2012). Association of particulate air pollution with daily mortality: The China Air Pollution and Health Effects Study. Am. J. Epidemiol..

[6] Pascal M., Corso M., Chanel O., Declercq C., Badaloni C., Cesaroni G., Medina S. (2013). Assessing the public health impacts of urban air pollution in 25 European cities: Results of the Aphekom project. Sci. Total Environ..

[7] Katsouyanni K., Touloumi G., Samoli E., Gryparis A., Le Tertre A., Monopolis Y., Rossi G., Zmirou D., Ballester F., Boumghar A. (2001). Confounding and Effect Modification in the Short-Term Effects of Ambient Particles on Total Mortality: Results from 29 European Cities within the APHEA2 Project. Epidemiology.

[8] Dominici F., Peng R.D., Zeger S.L., White R.H., Samet J.M. (2007). Particulate air pollution and mortality in the United States: Did the risks change from 1987 to 2000?. Am. J. Epidemiol..

[9] Woodruff T., Darrow L., Parker J. (2007). Air pollution and postneonatal infant mortality in the United States, 1999–2002. Environ. Health Perspect..

[10] Carlin B.P., Gelfand A.E., Banerjee S. (2014). Hierarchical Modeling and Analysis for Spatial Data.

[11] Cressie N., Wikle C.K. (2015). Statistics for Spatio-Temporal Data.

[12] Sahu S.K., Bakar K.S. (2012). A comparison of Bayesian models for daily ozone concentration levels. Stat. Methodol..

[13] Bakar K.S., Sahu S.K. (2015). spTimer: Spatio-temporal bayesian modelling using R. J. Stat. Softw..

[14] Del S.S., Ranalli M.G., Bakar K.S., Cappelletti D., Moroni B., Crocchianti S., Salvatori R. (2016). Bayesian Spatiotemporal Modeling of Urban Air Pollution Dynamics. Top. Methodol. Appl. Stat. Inference.

[15] Lawson A.B., Choi J., Cai B., Hossain M., Kirby R.S., Liu J. (2012). Bayesian 2-stage space-time mixture modeling with spatial misalignment of the exposure in small area health data. J. Agric. Biol. Environ. Stat..

[16] Liang D., Kumar N. (2013). Time-space Kriging to address the spatiotemporal misalignment in the large datasets. Atmos. Environ..

[17] James S.H., Brian J.R. (2010). Adding spatially-correlated errors can mess up the fixed effect you love. Am. Stat..

[18] Lee H., Kim H., Honda Y., Lim Y.H., Yi S. (2013). Effect of Asian dust storms on daily mortality in seven metropolitan cities of Korea. Atmos. Environ..

[19] Son J.Y., Lee J.T., Kim H., Yi O., Bell M.L. (2012). Susceptibility to air pollution effects on mortality in Seoul, Korea: A case-crossover analysis of individual-level effect modifiers. J. Expo. Sci. Environ. Epidemiol..

[20] Microdata Integrated Service Home Page. https://mdis.kostat.go.kr.

[21] Pun K., Seigneur C. (2006). Using CMAQ to Interpolate among CASTNET Measurements.

[22] Choi J., Lawson A.B. (2018). A Bayesian two-stage spatially dependent variable selection model for space–time health data. Stat. Methods Med. Res..

[23] Besag J. (1974). Spatial interaction and the statistical analysis of lattice systems. J. R. Stat. Soc. Ser. B Stat. Methodol..

[24] Knorr-Held L. (2000). Bayesian modelling of inseparable space-time variation in disease risk. Stat. Med..

[25] Bowman D.D., Liu Y., McMahan C.S., Nordone S.K., Yabsley M.J., Lund R.B. (2016). Forecasting United States heartworm Dirofilaria immitis prevalence in dogs. Parasites Vectors.

[26] MRC Biostatistics Unit Home Page. https://www.mrc-bsu.cam.ac.uk/software/bugs/the-bugs-project-winbugs/.

[27] Gelman A., Rubin D.B. (1992). Inference from iterative simulation using multiple sequences. Stat. Sci..

[28] The R Project for Statistical Computing Home Page. https://www.r-project.org.

[29] Park A., Hong Y., Kim H. (2011). Effect of changes in season and temperature on mortality associated with air pollution in Seoul, Korea. J. Epidemiol. Community Health.

[30] Pope C.A., Burnett R.T., Thun M.J., Calle E.E., Krewski D., Ito K., Thurston G.D. (2002). Lung cancer, cardiopulmonary mortality, and long-term exposure to fine particulate air pollution. Jama.

[31] Jerrett M., Burnett R.T., Ma R., Pope C.A., Krewski D., Newbold K.B., Thurston G., Shi Y., Finkelstein N., Calle E.E. (2005). Spatial analysis of air pollution and mortality in Los Angeles. Epidemiology.

[32] World Health Organization (2000). Quantification of Health Effects of Exposure to Air Pollution: Report on A WHO Working Group.

[33] Kan H., London S.J., Chen G., Zhang Y., Song G., Zhao N., Jiang L., Chen B. (2008). Season, sex, age, and education as modifiers of the effects of outdoor air pollution on daily mortality in Shanghai, China: The Public Health and Air Pollution in Asia (PAPA) Study. Environ. Health Perspect..

[34] Burnett R.T., Brook J., Dann T., Delocla C., Philips O., Cakmak S., Vincent R., Goldberg M.S., Krewski D. (2000). Association between particulate-and gas-phase components of urban air pollution and daily mortality in eight Canadian cities. Inhal. Toxicol..

