# Supplementary material for: Space–Time Relationship between Short-Term Exposure to Fine and Coarse Particles and Mortality in a Nationwide Analysis of Korea: A Bayesian Hierarchical Spatio-Temporal Model
Source: Int J Environ Res Public Health. 2019 Jun 14;16(12):2111. doi: 10.3390/ijerph16122111 (PMC6617003; doi:10.3390/ijerph16122111)
Supplement: Supplementary file 1 [file ijerph-16-02111-s001.pdf]

International Journal of
Environmental Research and Public Health

MDPI

Supplementary material

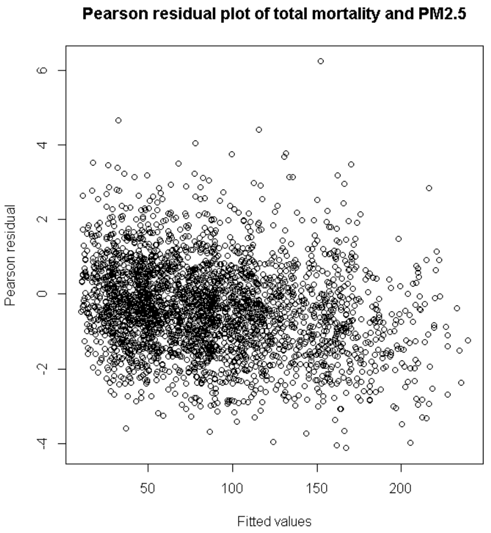

(a) total mortality and $PM_{2.5}$

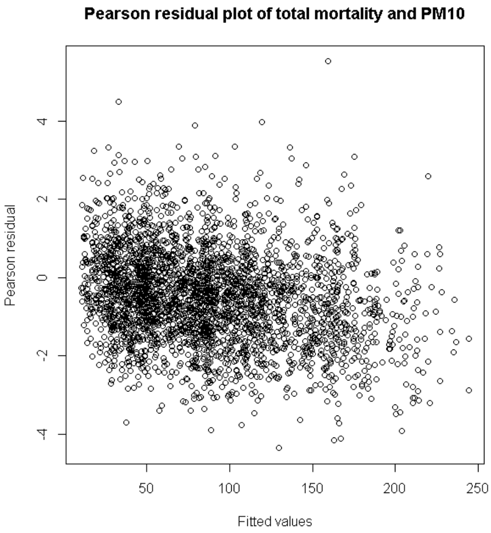

(b) total mortality and PM_{10}

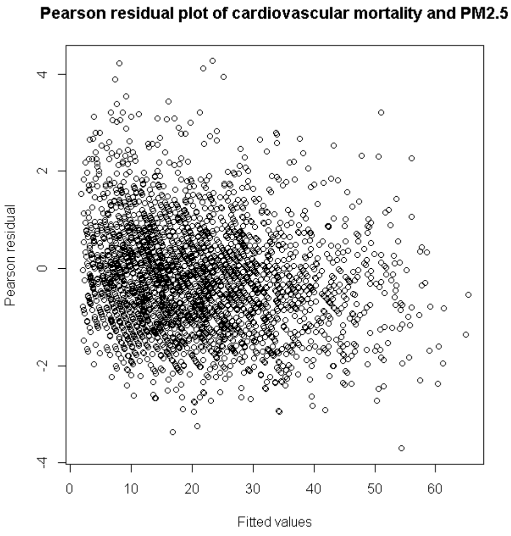

(c) cardiovascular mortality and $PM_{2.5}$

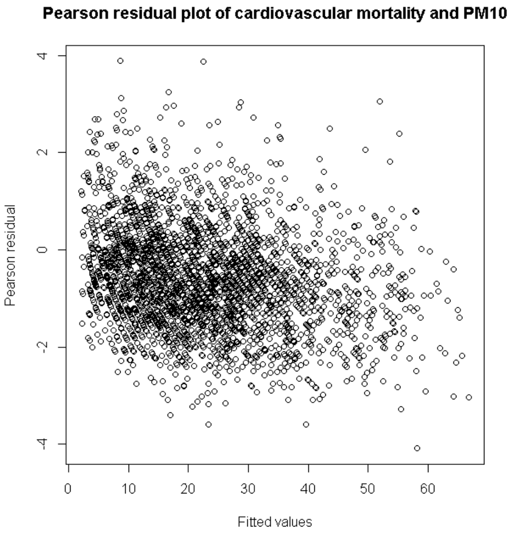

(d) cardiovascular mortality and PM_{10}

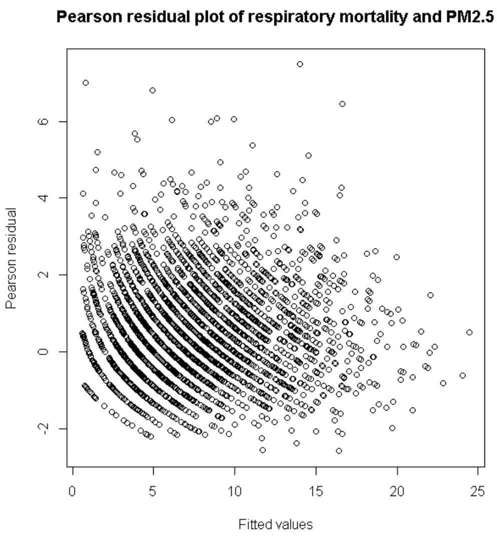

(e) respiratory mortality and $PM_{2.5}$

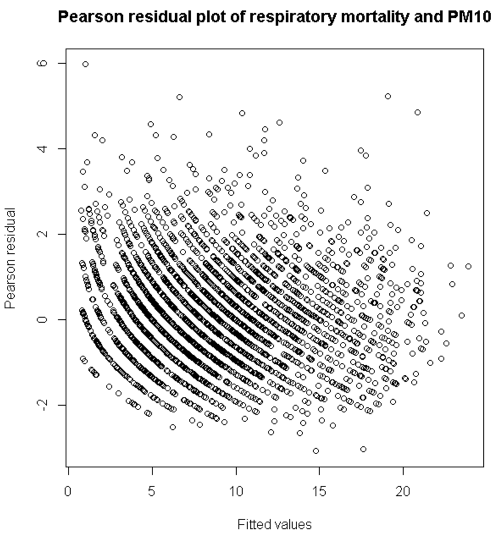

(f) respiratory mortality and PM_{10}

Figure S1. Plots of Pearson residuals versus the forecasted values for different types of mortality and air pollutants.

Figure S1 shows the plots of Pearson residuals. The x-axis indicates the forecasted death counts for 2015 and y-axis indicates the Pearson residuals of the observed death counts and forecasted death counts. Pearson residuals r_i are calculated using the following formula:

$$r_i = \frac{y_i - \widehat{y_i}}{\sqrt{\widehat{y_i}}} \text{ where } y_i \; is\ the\ observed\ death\ count\ and\ \widehat{y_i}\ is\ the\ forecasted\ death\ count.$$
